# Exploiting a Reference Genome in Terms of Duplications: The Network of Paralogs and Single Copy Genes in *Arabidopsis thaliana*

**DOI:** 10.3390/biology2041465

**Published:** 2013-12-09

**Authors:** Mara Sangiovanni, Alessandra Vigilante, Maria Luisa Chiusano

**Affiliations:** 1 Department of Electrical Engineering and Information Technology, University of Naples Federico II, Napoli 80125, Italy; E-Mail: mara.sangiovanni@unina.it; 2 Department of Genetics, Evolution and Environment, UCL Genetics Institute, University College London, London, WC1E 6BT, UK; E-Mail: a.vigilante@ucl.ac.uk; 3 Department of Agricultural Sciences, University of Naples Federico II, Napoli 80055, Italy

**Keywords:** gene duplication, paralog genes, single copy genes, singleton, gene network, *Arabidopsis*, genome annotation

## Abstract

*Arabidopsis thaliana* became the model organism for plant studies because of its small diploid genome, rapid lifecycle and short adult size. Its genome was the first among plants to be sequenced, becoming the reference in plant genomics. However, the *Arabidopsis* genome is characterized by an inherently complex organization, since it has undergone ancient whole genome duplications, followed by gene reduction, diploidization events and extended rearrangements, which relocated and split up the retained portions. These events, together with probable chromosome reductions, dramatically increased the genome complexity, limiting its role as a reference. The identification of paralogs and single copy genes within a highly duplicated genome is a prerequisite to understand its organization and evolution and to improve its exploitation in comparative genomics. This is still controversial, even in the widely studied *Arabidopsis* genome. This is also due to the lack of a reference bioinformatics pipeline that could exhaustively identify paralogs and singleton genes. We describe here a complete computational strategy to detect both duplicated and single copy genes in a genome, discussing all the methodological issues that may strongly affect the results, their quality and their reliability. This approach was used to analyze the organization of *Arabidopsis* nuclear protein coding genes, and besides classifying computationally defined paralogs into networks and single copy genes into different classes, it unraveled further intriguing aspects concerning the genome annotation and the gene relationships in this reference plant species. Since our results may be useful for comparative genomics and genome functional analyses, we organized a dedicated web interface to make them accessible to the scientific community

## 1. Introduction

*Arabidopsis thaliana* has been used as the model organism for molecular studies in plant biology [1,2,3,4] since 1970. As a consequence, the establishment of several resources made this organism the first plant having its small diploid genome completely sequenced [5]. Furthermore, as sequencing technologies grew easier and less expensive, *Arabidopsis* has achieved an increasing importance as a reference in plant comparative genomics [6,7,8,9,10,11,12,13,14]. A reference genome should be exhaustively annotated and well understood in terms of evolutionary history [15], whereas *Arabidopsis* still shows an unclarified complexity in terms of the events that molded its genome organization [12,16,17,18,19]. The *Arabidopsis* genome appears to have undergone distinct ancient whole genome duplication events, followed by gene reduction and diploidization [18,20,21,22,23,24,25,26,27,28,29,30]. Moreover, what dramatically increases its complexity are multiple rearrangements (*i.e.*, deletions, inversions, translocations), which duplicated, relocated and split up the retained portions [21,31,31,32,33], together with probable chromosome reductions within the Brassicaceae family [32,34,35]. Supporting evidence of a deep reshuffling of the *Arabidopsis* gene content has been revealed since the first release of the genome [5] and was further highlighted by recent studies based on homology investigations among plant species [16,36,37].

Over the last ten years, several studies aimed at clarifying the evolutionary history of *A. thaliana* [18,19,38,39,40,41,42,43]. They provide, often based on unrelated efforts, several distinct, but not always comparable, sets of paralogs or singleton genes. These collections are rather heterogeneous, due to the numerous methodologies used to achieve different, but closely-related, aims, such as the identification of paralogs [18,19,21,25,26,40] and/or singletons [44], or aging, evolution [45] and family organization [46] analyses. However, a common methodological framework widely accepted as a gold-standard is still missing. This may lead to non-reproducible, incomparable and sometimes discordant results, further making difficult their interpretation and exploitation. Though most of the implemented approaches are based on sequence comparisons using either FASTA (FAST-Alignment) [47,48,49,50] or BLAST (Basic Local Alignment Search Tool) based tools [51,52,53,54,55], often, there is no general agreement in terms of thresholds and settings to take into account [56], and therefore, results are not immediately comprehensible. GreenPhyl[44], Ensembl compara [57] and Plaza [45] are three examples of widely accepted platforms used as plant resources for duplicated gene collections, and consequently, they also provide information on singleton genes. However, these resources have heterogeneous results, thus making a unified view of the data organization difficult to achieve. Here, we present a complete pipeline, based on well-known bioinformatics approaches [58,59], enriched with details on the settings and on the specific methodological approaches involved in the definition and classification of paralogs and single copy genes, addressing the main controversial issues that may strongly affect the results, their quality and reliability Besides providing a contribution to the definition of a widely-accepted strategy for reproducible results, we present a coherent classification of the *A. thaliana* genes that may support similar studies and comparative genomics. On the other hand, our approach revealed still intriguing aspects concerning the gene annotation of *A. thaliana* that are worthwhile to be considered by the interested scientific community.

## 2. Methods

### 2.1. Data Retrieval

The *Arabidopsis thaliana* genome (release TAIR9, June, 2009) was downloaded from the TAIR website [60]. TAIR9 consists of 27, 379 protein-coding genes, 4, 827 pseudogenes or transposable elements and 1, 312 ncRNAs. We considered only the 27, 169 nuclear protein-coding genes and the corresponding transcript sequences. Mitochondria and chloroplast genes were excluded from the analyses. Moreover, we grouped as *non-protein-coding genes* the pseudogenes, the small nuclear RNAs (snRNAs), the small nucleolar RNAs (snoRNAs), the micro RNAs (miRNAs), the transfer RNAs (tRNAs), the ribosomial RNAs (rRNAs), the other RNAs, and the transposable elements. All these RNAs were classified according to the locus type assigned by TAIR. Intergenic regions were also downloaded from the TAIR website. The complete set of *A. thaliana* Expressed Sequence Tag (EST) sequences was downloaded from the GenBank release of 8 April 2010. *Arabidopsis* gene family information was downloaded from the TAIR website. To enrich the list of gene families, we considered the non-redundant collection obtained from transcription factor families reference databases [61,62].

### 2.2. BLASTp Analysis

All-against-all protein sequence similarity searches were performed using the BLASTp (protein BLAST) program [63] considering different settings.

1. The Expect-value cut-off. To detect different levels of sequence similarity among protein-coding genes, two different Expect-value (E-value, *E*) cutoffs were used: a more stringent threshold (*E* ≤ 10^−10^) and a less stringent one (*E* ≤ 10^−5^) [64,65]. To ensure the correct definition of singleton genes as those not having similar copies in the genome, we considered an even looser E-value threshold (*E* ≤ 10^−3^).
2. The Rost's formula. To determine whether two proteins in a genome are paralogs when the similarity between them is in the so-called twilight zone (20%–30% of identity/length ratio), the Rost's formula [56,58] was applied with the cut-off threshold set to *n* = 5, according to Li *et al.*, [59]. The use of this formula implies that all the alignments with a length shorter than 150 amino acids and with an identity score lower than 30% were discarded. These genes were removed both from the networks and from the singleton analysis, due to the intrinsic ambiguity of their paralogy relationships.
3. The low-complexity BLAST filter. The BLAST analysis applies by default a masking of low-complexity regions in the query sequence [66]. Indeed, because of their repetitive nature, low-complexity regions, which are very abundant in biological sequences, may result in biologically meaningless high scoring hits [67]. However, often, sequence similarities can be missed using the masking of low complexity regions, in particular in the case of small sequences. Therefore, after a first BLASTp analyses with the default parameters, both using the more stringent cut-off and the less stringent one, another BLASTp analysis (*E* ≤ 10^−5^) was performed without the masking filter, to identify genes that could have been masked hiding similarities to other genes. Sequences showing similarity when unmasked were not included in the subsequent analyses, since they cannot be considered as singletons and their similarity with other proteins may not be necessarily associated with a paralogy relationship.

### 2.3. Detection of Differences in ORF Assignments

To check for possible similarities hidden by different open reading frame (ORF) assignments, *Arabidopsis* transcript sequences were aligned *vs*. the *Arabidopsis* protein collection with a BLASTx analysis (*E* ≤ 10^−5^).

### 2.4. Identification of the Similarities of Protein Coding vs. Non-Protein-Coding Regions

In order to consider nucleotide-based similarities of protein-coding genes with other regions of the entire genome, the full-length genes (queries) were searched against both non-protein-coding genes and intergenic regions, using the nucleotide BLAST (BLASTn) tool (*E* ≤ 10^−5^). The resulting alignments were manually curated considering the intron-exon structure of the query: in particular, we distinguished alignments involving only intronic regions of the query and alignments involving coding regions of the query and covering at least half of its length. To determine any further significant sequence similarity of a protein coding gene within the genome, a BLASTn analysis (*E* ≤ 10^−5^) was performed, searching against all the *Arabidopsis* intergenic regions.

### 2.5. EST-Based Functional Validation of Singleton Genes

We confirmed the functionality of the genes by looking for EST evidence. Transcript sequences from singleton genes were aligned *vs*. ESTs (with a BLASTn, no E-value cutoff) [63]. This permitted us to define transcripts without any similarity with EST sequences. We then considered the statistical significant alignments with *E* ≤ 10^−5^. However, the E-value threshold itself cannot be enough to confirm the association of a transcript to an EST. Since ESTs represent *sequence tags* of a transcript, we generally expected an EST sequence to be shorter than the transcript length, but this was not always the case. Therefore, we imposed further criteria to validate the alignments: we considered match lengths greater or equal to 60% of the shortest sequence between the two alignment (*i.e.*, the transcript or the EST). An EST sequence should be 100% identical to the transcript in the region of a match, though 95% of the identity should better ensure the consideration of possible sequencing errors (typical in EST sequencing). We decided to consider a cutoff at 90% identity This also permits us to strictly determine those transcripts that cannot be reliably associated with ESTs when showing an alignment score lower than the cutoff. Alignments with a scoring higher or equal to the cutoff were analyzed in terms of the relative length of the transcript and the corresponding best matching EST. We defined the parameter, delta, as the EST length minus the transcript length, to verify if the EST is shorter or equal to the transcript length (Δ ≤ 0), as it should be. In the case of positive deltas, we accepted those alignments with Δ ≤ 20 nucleotides, since we assumed that the presence of an EST longer than the transcript of a maximum of 20 nucleotides can be ascribed to EST regions not trimmed and/or still containing contaminations.

RNA-sequencing (RNA-seq) data were used to further verify the expression of these singletons. We based our analyses on a multiple tissue RNA sequencing experiment of two *A. thaliana* accessions (GSE30795), downloaded from the Gene Expression Omnibus (GEO) database [68]. These data were generated with the Illumina Genome Analyzer IIx from messenger RNA isolated from seedling, root and floral bud tissues. Isoform abundance quantifications, *i.e.*, RPKM (Reads per Kilo Base per Million) values, were also downloaded. An RPKM threshold of one was applied to consider a gene/transcript as expressed in at least one tissue, as in [69].

### 2.6. Searching for Orthologs

To confirm our findings, we used the BioMart tool and the Ensembl plants database (plant Mart Release 5) [57,70] for the detection of ortholog genes among evolutionarily close (*Arabidopsis lyrata* and *Brassica rapa*) and distant (*Oryza sativa*, *Sorghum bicolor*, *Vitis vinifera* and *Populus trichocarpa*) plant species.

### 2.7. Network Definition

The network extraction process was based on considering the *Arabidopsis* protein-coding gene collection as an undirected graph with the vertex representing genes (nodes) and edges representing paralogies. Networks correspond to the connected components of the graph and were extracted with a recursive depth-first search on it.

### 2.8. The Web-Accessible Database

The website has been implemented with a PHP (Hypertext Preprocessor) [71] interface and a MySQL [72] open source database. The query system was designed to search by gene ID, annotation, gene class and network name. The network graphic representation is based on the Cytoscape tool [73].

## 3. Results

### 3.1. Identification of Duplicated Genes

**Figure 1:**
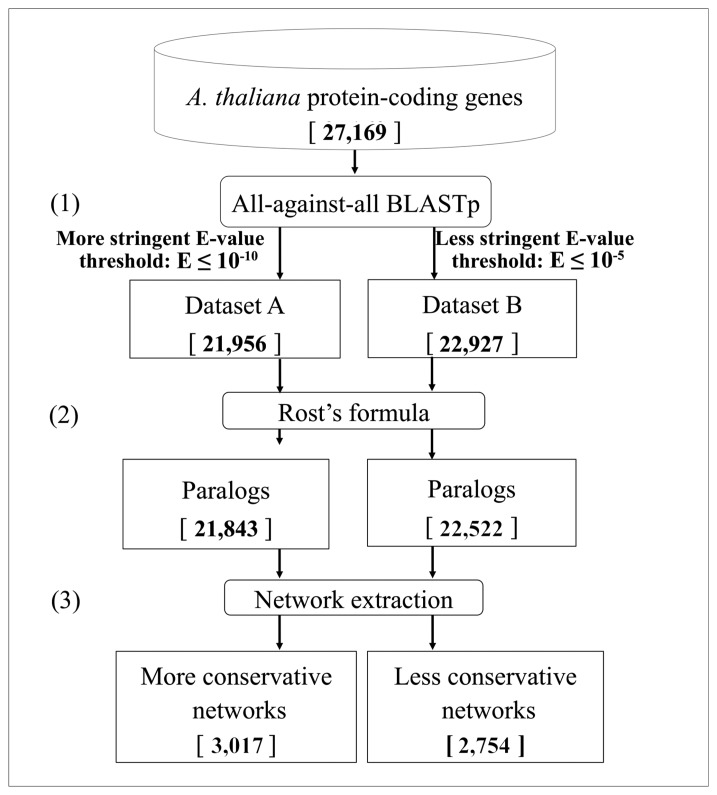
Pipeline for the identification of duplicated genes. Starting from the entire collection of 27, 169 *Arabidopsis* nuclear proteins, all-against-all BLASTp analyses were performed using two different parameter settings, producing two different datasets (**A**, **B**) (step **1**). The application of the Rost's formula produced two collections of genes with more reliable protein similarities (step **2**). Those genes, indicated as paralogs, were afterwards organized into networks (step **3**). The numbers resulting at each step are reported in square brackets.

We analyzed the whole *Arabidopsis* protein-coding gene collection (release TAIR9 [60]), and we identified the possible pair-wise paralogy relationships, obtaining an appropriate classification of the considered genes. In the first step of the pipeline (Figure 1), an all-against-all protein sequence similarity search was performed using the BLASTp program [63]. Two different E-value (*E*) cutoffs were used: a more stringent threshold (*E* ≤ 10^−10^) and a less stringent one (*E* ≤ 10^−5^) [64,65].

About 85% of the proteins have at least one significant sequence similarity *vs*. another protein in the collection (Figure 1, Datasets A and B, respectively). To detect significant paralogies, the Rost's formula [58,74] was used to distinguish reliable alignments in the two datasets among the ones falling in the so-called twilight-zone (20%–30% of identity). As a consequence, 405 out of 22, 927 and 113 out of 21, 956 genes, obtained using the two E-value cutoffs, respectively, were removed from the lists of genes sharing significant similarities. The remaining 22, 522 and 21, 843 genes of Datasets A and B (Figure 1) were therefore classified as *paralogs* and were afterwards organized into the so-called *networks of paralogs* (Figure 1, step 3).

### 3.2. Networks of Paralogs

For each E-value threshold, two distinct sets of networks were therefore obtained: the more conservative (*E* ≤ 10^−10^) and the less conservative (*E* ≤ 10^−5^) ones. The first set consists of 3, 017 networks, while the second set consists of 2, 754 ones (Figure 1).

Each gene belongs to one and only one network and is connected by at least one paralogy relationship to another gene in the same network. It is worth noting that although Dataset A contains fewer genes showing paralogies than Dataset B, the number of extracted networks is higher. This is due to the more stringent filtering applied to obtain Dataset A. The higher number of networks indeed indicates the detection of fewer paralogies among the genes included in Dataset A.

**Figure 2:**
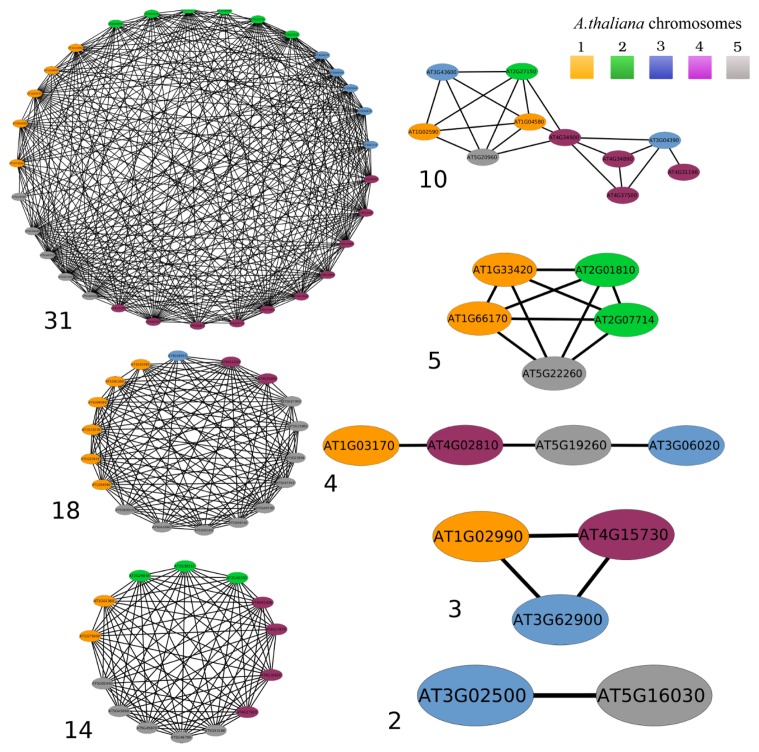
Examples of size and complexity exhibited by the networks. Each vertex is a gene (node), with colors associated with the five *Arabidopsis* chromosomes according to the legend. Black lines represent paralogies between genes. Numbers near each network indicate the size in terms of the number of included genes.

The networks show differences, both in their size (*i.e.*, the number of included genes) and in their complexity (*i.e.*, the number of paralogy relationships), as shown in the examples in Figure 2.

Table 1 summarizes the number of networks per size distribution together with the number of genes they include, for both sets. About 30% of the genes are distributed in small networks, while 13% of duplicated genes belong to medium-sized networks. Larger networks contain about 15% of the genes. Only one network contains more than 210 genes in both sets, including more than 20% of the entire collection. One-hundred and sixteen out of 2, 754 networks defined using the less conservative threshold disappear when the more stringent threshold is applied, since they contain genes that all become singletons. Two-thousand four-hundred and ten 2,410 networks stay unchanged, whereas the remaining ones were split into smaller distinct networks. This confirms the usefulness of keeping results from both thresholds.

**Table 1:** Number of networks, size distribution and number of included genes for each E-value threshold.

**Network Size**	**Less conservative networks** **(*E*** ≤ **10**^−^**^5^)**		**More conservative networks** **(*E*** ≤ **10**^−^**^10^)**	
	
	**Network Number**	**Gene Number**	**Network Number**	**Gene Number**
2	1, 215	2, 430	1, 347	2, 694
3 to 9	1, 232	5, 966	1, 370	6, 217
10 to 30	241	3, 421	216	3, 603
31 to 209	65	3, 871	83	4, 161
> 210	1	6, 834	1	5, 168

The complexity of a network and the correspondent size are both important to understand structural and evolutionary relationships among the genes it includes. A network can be fully connected, this indicating the presence of paralogy relationships among all the genes (networks of sizes 5 and 3, Figure 2). In this case, considering n the number of nodes, $\frac{n(n-1)}{2}$ edges should be present. In general, the minimum number of paralogies in a network is *n* − 1, as shown for the network of size 4 in Figure 2, where each gene has at most two paralogs. The investigation of the networks organization in terms of complexity may not be immediate, especially when the network size increases. In this frame, the largest networks obtained using both the E-value cutoffs (Table 1) represent interesting case studies. For example, the largest more conservative network made of 5, 168 genes (Table 1) includes 287, 780 paralogies. This number is far from the expected maximum (more than 13 million paralogies). Nevertheless, it does not provide any hint on the structural features of the network nor on the relationships among the included genes. Therefore, we investigated the complexity of this network in terms of the number of paralogies per gene. Only 448 genes resulted in having a low number of paralogies (from one to four) (Table 2). In order to unravel this network and get further information, we subsequently removed the genes having up to two, three and four paralogies. Both the number of removed genes at each step and the size for all the resulting networks are shown in Table 3.

The removal of genes always results in one large network, including about 85% of the genes. The presence of a network core that cannot be split into smaller sub-networks reveals that 19% of the *Arabidopsis* protein-coding genes are in someway extensively structurally related.

**Table 2:** Distribution of genes per number of paralogies in the biggest more conservative network.

**Number of paralogies**	**Number of genes**
1	92
2	118
3	118
4	120
5 to 10	491
11 to 50	1, 292
51 to 200	1, 804
>200	1, 133

**Table 3:** More conservative largest network organization investigated by the removal of paralogy relationships.

**Removed paralogies**	**Up to 2**	**Up to 3**	**Up to 4**
**Removed genes**	**211**	**328**	**448**
	4, 766	4, 658	4, 197
	169	138	157
	13	28	134
	5	13	95
Resulting network sizes			90
			28
			18
			9
			7

### 3.3. Two-Gene Networks

The 10% of the protein-coding genes are organized in networks, including only two genes (*two-gene networks*). The amount of genes belonging to two-gene networks per chromosome and their mutual distribution among the upper (U) and lower (L) arms (defined according to [75]) are summarized in Table 4 for the less conservative E-value cutoff (similar results holds for the more conservative cutoff). The maximum number of two-gene networks shared per arm pair is 107 (between upper and lower arms of chromosome 1).

**Figure 3:**
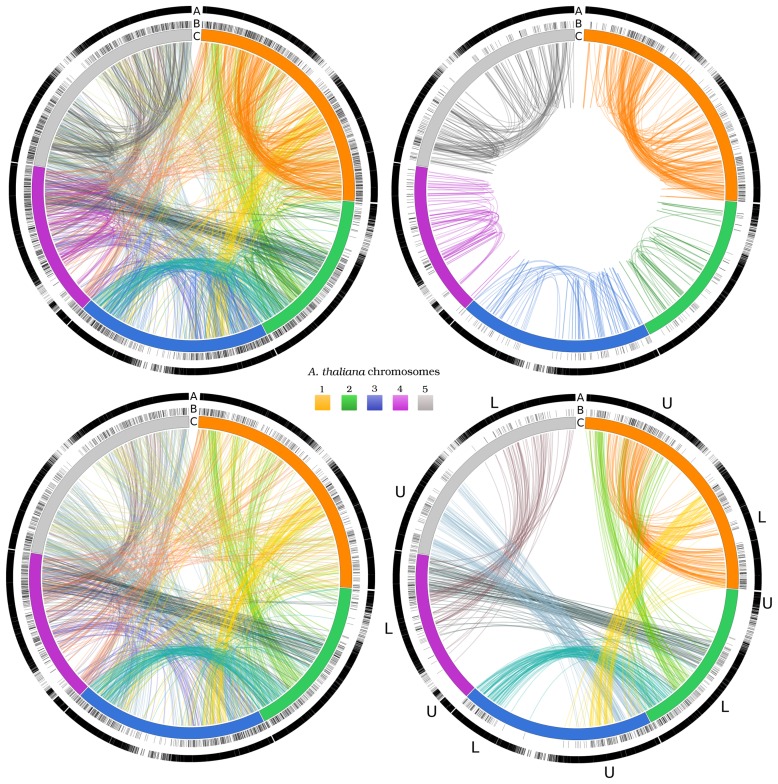
Chromosome distribution of two-gene networks. The *A. thaliana* chromosomes are indicated in circle C as curved bars with a length proportional to the number of base pairs and colors according to the legend. Circle A indicates the protein coding gene distribution along the chromosomes. The lines linking chromosome regions indicate paralogies (two-gene networks). The images show: all two-gene networks (top left); intra-chromosome two-gene networks; (top right); inter-chromosome two-gene networks (bottom left). The bottom right image shows the seven largest groups of paralogies shared between upper (U) and lower (L) chromosome arms (colors are as in Table 4). Circle B indicates the distribution of those genes involved in the represented paralogies. Data are shown only for a less conservative E-value threshold (*E* ≤ 10^−5^).

The table also highlights (in bold) the topmost seven values, obtained using a threshold of 55, *i.e.*, half of the resulting maximum value. The use of this threshold highlights the most relevant inter- and intra-chromosome patterns. The paralogy distributions for two-gene networks are also shown in Figure 3, where, besides the overall gene distribution (top left image), intra- and inter-chromosome relationships are separately depicted (top right and bottom left images, respectively). Intra-chromosome duplications are clearly visible (top left image), in particular between the upper and the lower arms of chromosome 1 and chromosome 5. Inter-chromosome relationships (bottom left image) are better highlighted when the topmost values from Table 4 are considered (bottom right image). The two-gene networks emphasize the presence of conserved patterns of duplicated gene pairs in evident syntenic arrangement, that may provide a key element in unraveling the evolutionary mechanisms that shaped the *Arabidopsis* genome organization, in the frame of the proposed whole genome duplications and subsequent diploidization, reshuffling and reduction events [18,20,21,25,26,27,28,30,31,39,40,41].

**Table 4:** Distribution of two-gene networks among chromosomes. For each chromosome (CHR), the number of genes involved in a two-gene network and the total number of protein-coding genes are indicated (second row). The amount of two-gene networks linking the chromosomes arms (U: upper; L: lower) is shown. Values greater than half of the maximum (55) are in bold.

		**CHR1**		**CHR 2**		**CHR 3**		**CHR 4**		**CHR 5**	

		**714/7,054**		**394/4,237**		**561/5,436**		**401/4,124**		**624/6,318**	
CHR 1	U	23	**107**	5	**62**	21	18	14	34	18	28
	L	**107**	33	4	21	**61**	17	4	25	19	37
CHR 2	U	5	4	5	1	3	0	0	3	2	6
	L	**62**	21	1	30	21	**81**	2	**68**	24	20
CHR 3	U	21	**61**	3	21	28	16	7	37	**86**	29
	L	18	17	0	**81**	16	8	5	12	22	37
CHR 4	U	14	4	0	2	7	5	2	6	7	5
	L	34	25	3	**68**	37	12	6	43	19	**57**
CHR 5	U	18	19	2	24	**86**	22	7	19	35	39
	L	28	37	6	20	29	37	5	**57**	39	30

### 3.4. Exploiting Networks: Analysis of Arabidopsis Gene Families

The construction of networks of paralogs also provides support for the study of *Arabidopsis* gene families. Six-thousand six-hundred and forty-seven genes are classified into gene families on the TAIR website [60]. Enriching this list using the annotation of transcription factor families from reference databases [61,62,76], we obtained a total of 7, 573 family-associated protein-coding genes belonging to 380 different gene families. The remaining 19, 569 genes are defined as family-not-associated genes. Ninety-seven percent of family-associated genes belong to networks. The distribution of the members of each gene family among the networks of paralogs (in both the more and the less conservative networks) may highlight still undefined associations. We classified about 70% of the gene families, defining them as *exclusive* families, since they have all the members arranged into one network, confirming the reliability of the networks' organization for the classification of paralogs. The other 30% of the families are instead classified as *split* families, since their members are distributed among different networks or are classified as singletons. Hence, networks containing only one family are classified as *exclusive* networks, to distinguish them from the *mixed* ones, *i.e.*, those containing genes belonging to different families and/or family-not-associated genes (Table 5).

**Table 5:** Distribution of family-associated genes in the networks of paralogs at the different thresholds.

	**Less conservative networks** **(*E*** ≤ **10**^−^**^5^)**	**More conservative networks** **(*E*** ≤ **10**^−^**^10^)**
Exclusive families in exclusive networks	52	58
Exclusive families in mixed networks	227	212
Split families in exclusive networks	20	29
Split families in mixed networks	81	81

**Figure 4:**
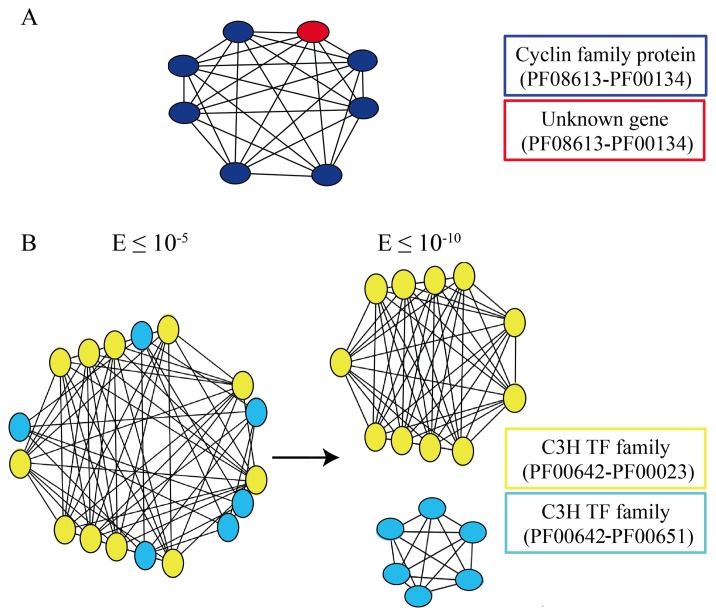
Use of networks of paralogs to investigate gene families and gene annotation. (A) all the genes in the network belonging to the same gene family (the Cyclin family) are in blue. The red gene is annotated as *unknown*. Paralogy relationships between members of the network are supported by the sharing of the same protein domains. (**B**) the less conservative network (1) is made of genes belonging to two different families, the transcription factor family C3H (yellow) and the BTB/POZ family (blue). When the more stringent E-value cutoff is used, the network is split into two smaller networks (2 and 3), each one containing only genes belonging to the same family.

Split families represent heterogeneous families, and their organization in exclusive networks indicates a self-consistent structure similarity of sub-groups of the family, which is often confirmed by the classification available from TAIR [60]. The split families, when organized into mixed networks, can be associated with members of other families, as well as genes without a clear annotation (*unknown genes*); see Figure 4 (panel A).

Interestingly, 2, 787 out of 5, 305 genes still annotated as coding for “unknown proteins”, belong to networks (with the less stringent threshold). This means that their functional annotation can be improved thanks to the relationships with other members of the same network.

The use of at least two different E-value cutoffs, as proposed here, also provides further information on the structural peculiarities of the families. As shown in Figure 4 (panel B), members of different families are contained in the same network when the (*E* ≤ 10^−5^) threshold is used, while they are split into two distinct exclusive ones, when the more conservative threshold (*E* ≤ 10^−10^) is applied.

This strategy can be extended to improve the gene family classification for a species annotation, promoting the identification of novel families and the annotation of family-not-associated genes, as well as of genes annotated as *unknown*. The use of the domains from the PFAM (Protein Families) database [77] confirms paralogy relationships either between genes from the same family or from different families in mixed networks (data not shown) or with non-family-associated genes.

### 3.5. Classification of Non-Paralog Genes and Identification of True Singletons

**Figure 5:**
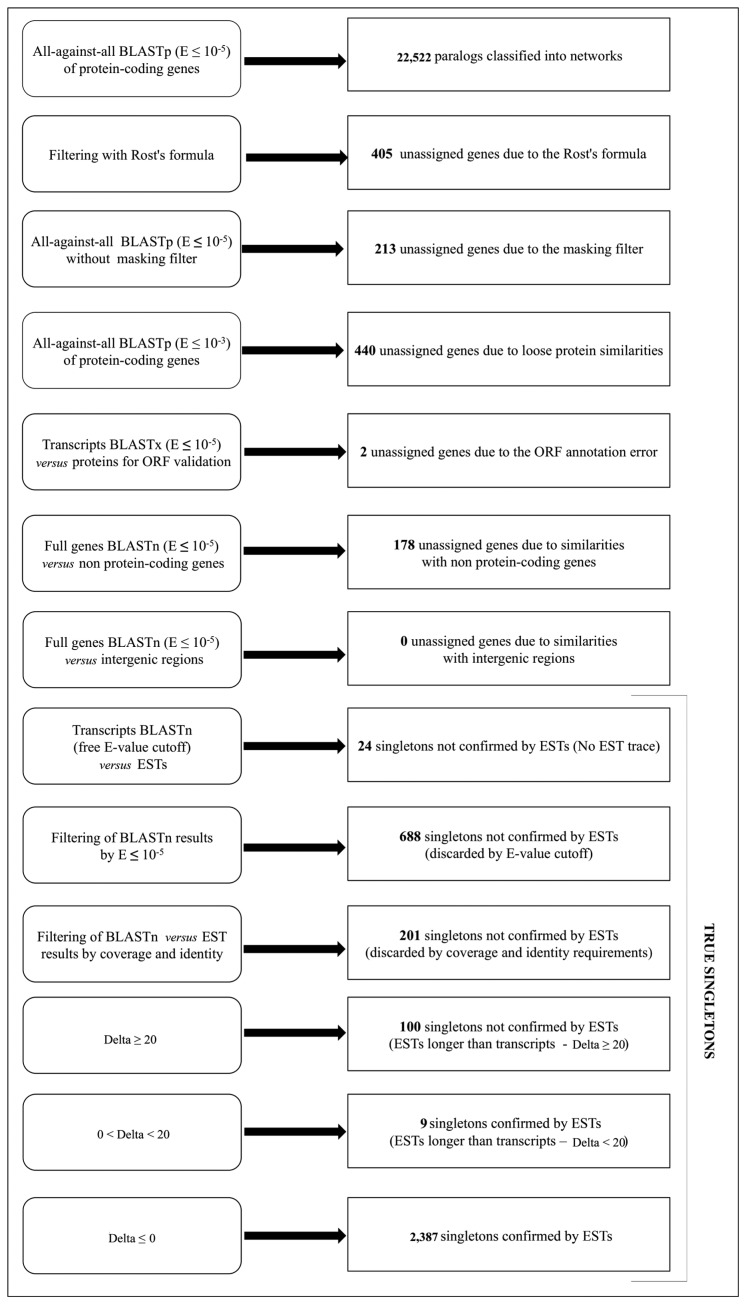
Classification of the *Arabidopsis* protein-coding gene collection. A summary of the performed analyses is indicated here together with the obtained classes and the number of genes herein. EST, Expressed Sequence Tag.

The presence of *Arabidopsis* genes having no statistically significant protein sequence similarity with other nuclear protein-coding genes was further investigated, also to define a suitable strategy for the detection of those genes that we will eventually call true singletons, *i.e.*, genes without any similarity *vs*. the entire genome sequence. We defined as *paralogs* those genes having at least one duplicate when using the less stringent E-value cutoff of *E* ≤ 10^−5^ (Figure 1). All the remaining genes were classified considering the analyses listed in Figure 5. When the Rost's formula was applied (Figure 1, step 2), 405 genes were excluded from the dataset of duplicated genes and were classified as *unassigned genes due to the Rost's formula*. A second BLASTp analysis at *E* ≤ 10^−5^ was performed removing the filter for the masking of low complexity regions, in order to identify potential paralogy relationships involving masked genes. The resulting 213 genes showing significant similarity when removing the masking filter were neither included in the paralogs class, nor considered as singletons, and were therefore classified as *unassigned genes due to the masking filter*. We considered the E-value cut-off at *E* ≤ 10^−5^ as the upper limit to define reliable protein sequence similarities (Figure 1, step 1) [78]. However, in order to exclude the loose similarities among proteins encoded in the genome, a BLASTp analysis with the E-value cutoff *E* ≤ 10^−3^ was applied, and 440 genes showing similarities with other proteins were classified as *unassigned genes due to loose protein similarity* thanks to this new setting.

The *Arabidopsis* transcript sequences were aligned *vs.* the protein collection using a BLASTx analysis (*E* ≤ 10^−5^) [63] to identify nucleotide similarities that can be hidden by different ORF assignments. Indeed, in one case, we confirmed the paralogy relationship between the two genes belonging to the MT1 family (AT1G07600 and AT5G56795), otherwise undetectable at the protein level, due to an ORF misassignment of the MT1B gene (AT5G56795). The two genes were neither included in the paralogs class, nor considered as singletons, and were therefore classified as *unassigned genes due to ORF annotation error.* Once we explored the similarity among protein-coding genes, we further investigated the collection of the 3,587 still unclassified remaining genes to discard nucleotide-based similarities with other regions of the entire genome. The full-length genes (exon plus introns) were independently compared *vs.* both non-protein-coding genes and intergenic regions (BLASTn [63], *E* ≤ 10^−5^).

Specifically, 250 genes shared similarity with at least one *Arabidopsis* non-protein-coding gene. We took into account the intron-exon structure of the query to discard those matches involving exclusively the introns, and we considered as significant those alignments that involve at least one exon and that cover half of the entire mature transcript length. This resulted in 178 genes out of 250, accordingly classified as *unassigned genes due to similarities with non-protein-coding genes* (68 similar to transposon genes, 80 similar to pseudogenes, 29 similar to other RNA genes and 19 similar to more than one of the previous categories). No significant hits were found in the comparison of the 3,409 remaining genes *vs.* the intergenic regions, thus excluding the presence of duplicates in genome regions not annotated as genes. These protein-coding genes were defined as *true singletons*, since they share no similarity *vs.* the entire *Arabidopsis* genome sequence. Besides listing the analyses that determined the assignments of the entire protein-coding gene collection *of Arabidopsis* to different classes, Figure 5 also summarizes the class names we defined and the number of genes they include. The presence of singletons in a highly duplicated genome is a relevant issue to investigate gene retention mechanisms [79] and diploidization events [23]. Therefore, we decided to further investigate the 3,409 true singleton genes, assessing their functionality by exploiting Expressed Sequence Tag (EST) collections and investigating the presence of orthologs in other plant genomes.

### 3.6. True Singletons Validation Based on Expressed Sequence Tags

We confirmed the functionality of the genes by comparing the true singletons *vs.* the complete set of *A. thaliana* ESTs (with a BLASTn, no E-value cutoff) [63]. Among the 3,409 singletons, 24 transcripts did not find any match with ESTs and were classified as *singletons not confirmed by ESTs (no EST trace).*

We then considered the statistically significant alignments (*E* < 10^−5^). Six-hundred eighty-eight out of 3,409 were classified as *singletons not confirmed by ESTs (discarded by E-value cutoff)*, since the alignment of the correspondent transcripts with the ESTs did not satisfy the considered cutoff. However, to confirm the association of a transcript to an EST, alignments involving the remaining 2,697 genes were further investigated, considering parameters, such as the match length and the number of identities (see the Methods section). This analysis allowed us to classify 201 genes as *singletons not confirmed by ESTs (discarded by coverage and identity requirements)*. The remaining 2,496 alignments were analyzed in terms of the relative length of the transcript and the corresponding best matching EST using the Δ parameter. Considering the distribution of the alignments with positive Δ (data not shown), two classes are evident: one in which Δ ≤ 20 nucleotides and the other with Δ > 20 nucleotides. Nine alignments with Δ ≤ 20 nucleotides are considered as confirmed by ESTs, since the presence of an EST longer than the transcript of a maximum of 20 nucleotides can be ascribed to EST regions not trimmed and/or still containing contaminations. One-hundred transcripts have ESTs much longer than the annotated region and are therefore classified as *singletons not confirmed by ESTs (EST longer than the transcript).* Since the majority of the genes considered are described by only one isoform (only four of the 100 genes presented multiple transcripts), the observed matches with longer ESTs are not ascribable to misassigned ESTs and may therefore represent limited annotations of the entire gene structure. In conclusion, 1,013 transcripts were *singletons not confirmed by ESTs*. The expression of the unconfirmed true singleton genes was further validated using RNA-seq data from three different tissues (roots, seeds and floral bud). Using an RPKM threshold of one, we detected 818 out of 1,013 singleton genes not confirmed by ESTs as not expressed in any of these tissues. The remaining 195 genes show a low expression in one or more tissues (data not shown). We also confirmed by RNA-seq the expression of 2, 178 out of 2, 396 singletons confirmed by ESTs.

### 3.7. Orthologs of True Singleton Genes in Other Plant Species

The 3, 409 true singletons were analyzed to search for ortholog genes in two closely-related species (*Arabidopsis lyrata* and *Brassica rapa*) and in other plant species (*Oryza sativa*, *Sorghum bicolor*, *Vitis vinifera* and *Populus trichocarpa*).

The results are shown in Table 6, together with the EST analysis results. A total of 1, 449 singletons have orthologs in all the considered species, and they are all singletons confirmed by ESTs. Interestingly, no orthologs have been found for all 1, 013 singletons without ESTs confirmation, when considering evolutionary distant plant species. Only 56 of these singletons have orthologs in closely-related species. Our results suggest the need for further validation of these gene annotations.

**Table 6:** Orthologs of true singleton genes in two closely-related species (top) and other plant species at different evolutionary distance (bottom).

		**Singletons with orthologs in all the considered species**	**Singletons with orthologs in at least one species**+	**Singletons without orthologs**	**Total**
	EST confirmed	374	1,126	896	2,396
*A. Lyrata*	No ESTs	1	55	957	1,013
*B. Rapa*	**Totals**	**375**	**1,181**	**1,853**	**3,409**

*O. sativa*	EST confirmed	1,449	498	449	2,396
*P. trichocarpa*	No ESTs	0	0	1,013	1,013
*S. bicolor*	**Totals**	**1,449**	**498**	**1,462**	**3,409**
*V. vinifera*					

### 3.8. The Web-Accessible Database

We set up a web-based resource to make all the data so far described accessible to the scientific community [80]. The web resource permits users to obtain information for each *Arabidopsis* protein-coding nuclear gene and its classification according to Figure 5. Either the more or the less conservative networks of paralogs can be accessed and downloaded. Specifically, the web resource permits one to obtain detailed information for each *A. thaliana* nuclear protein coding gene at the two thresholds. If the gene is included in a network, the following information is provided: the list of direct paralogs (*i.e.*, the genes directly related to the selected gene by a paralogy relationship), the list of all the genes included in the network and the graphical representation of the network itself. The networks at the less stringent thresholds were named NETxGy_z, where NET stands for network and G for genes, x indicates the number assigned when sorting the total amount of networks by decreasing network size, y indicates the network size (*i.e.*, the number of included genes) and z is the number of networks or singletons in which the network is split when the more stringent cutoff is applied. This naming convention was established to (1) support the immediate understanding of the network structure and (2) help in associating the networks obtained at the two different thresholds. Please note that each gene belongs to one and only one network at each threshold. For genes without paralogs, the associated classification is reported. (See the help file on the website for more information.)

## 4. Discussion

The need for further investigations on the complexity of the *Arabidopsis thaliana genome* was evident since its first release, primarily for its role as a reference in plant comparative genomics [6,21,31,32,33,34,36,37,81]. To this aim, reviewing the organization of duplicated and single copy genes in *A. thaliana* is essential, though these approaches are not a novelty in genomics, as well as in plant genomics [44]. Indeed, several different bioinformatics resources are today available for *Arabidopsis* and other plant genomes [18,45,46,47,57], which include, for different purposes, information concerning gene duplications. However, the provided collections are quite heterogeneous and often hard to compare. This is mainly due to the lack of common frameworks concerning data sources, methods and details on the specific settings. Therefore, it is rather tricky to compare the methodologies and the results they produce, making it even more difficult to assess their exhaustivity, reliability and reproducibility. The methodologies are mainly based on BLAST supported approaches [51,52,53,63] with settings that have often, but independently, been discussed [27,44,56,58,66,67,74,82]. Here, we described all the details of a complete bioinformatics pipeline capable of detecting both duplicated and single copy genes in an annotated genome and its application to the analysis of the *A. thaliana* protein coding gene collection. The main results of the pipeline are lists of paralogs (including about 80% of the whole *Arabidopsis* protein-coding gene collection) and singleton genes (∼20%). The organization of paralogs into networks of genes provides a suitable support to their mining, their investigation and their management, to face both annotation and evolutionary issues. Indeed, as shown in Figure 4, analyses on networks of paralogs may contribute to the functional assignment of *unknown* genes, or to investigations on gene family relationships. Other evolutionary insights can also be inferred. As an example, one quarter of the genome has zero or at most only one paralog, which is rather intriguing in a widely duplicated genome [18,21,25,26,40,41]. Previous analysis on singleton genes in *Arabidopsis* reported their amount as being about 5, 000 genes [44]. Here, we obtained a dataset of 3, 409 true singletons. Moreover 29% of them could not be validated by ESTs. This last result and the evidence that most of the unconfirmed genes (957 out of 1, 013) has also no orthologs in both evolutionarily close and distant plant species highlights the need for further confirmations of the current annotation of a widely investigated reference genome. This is necessary for a better assessment of these genes and their evolutionary history in plants. Doubts of the quality of some gene annotation also arise from the identification of a mis-annotated open reading frame (ORF), which has never been highlighted in other *A. thaliana* related collections. Despite the relevance of the manifold resources already available [45,46,47,57] concerning gene duplication in *A. thaliana*, none of them reveals the relationship between the two metal ion binding MT1A (AT1G07600) and MT1B (AT5G56795) genes, which are paralogs. Indeed, these two genes are reported as singletons in all similar resources, since they are exclusively based on protein sequence analyses. The paralogy relationship that emerges from the nucleotide sequence similarities of proteins encoded on different frames, in fact, is only detectable by deeper investigations, such as the one here described, which expands the conventional protein-based paralog search to transcript analyses.

We also overviewed several methodological issues, addressing the main aspects that may strongly affect the results, their quality and reliability, trying to contribute to the definition of a widely accepted strategy for reproducible results. For example, most studies set one pre-defined E-value threshold to filter significant resulting alignments between proteins, in a BLASTp analysis. Indeed, it is well-known that the E-value cutoff is critical in the definition of whether genes are true paralogs [78,82]. Therefore, here, we selected two different thresholds, also useful for highlighting paralogies at different stringency between sets of genes. The less conservative threshold (*E* ≤ 10^−5^) provided a coarse-grained view of the gene distribution into networks, whereas a more stringent one (in our sample study, *E* ≤ 10^−10^) gave rise to reduced gene sets per network, separating those subsets sharing lower similarity. We also considered the presence of low-complexity regions [66,83], since they may cause non-homologous proteins to be unexpectedly similar or, *vice versa*, hide true similarities.

The detailed classification of the *A. thaliana* nuclear protein-coding gene collection and the whole bioinformatics pipeline description provide a unique comprehensive reference, to our knowledge, for similar efforts and for plant comparative genomics.

## 5. Conclusions

The list of duplicated genes, together with their organization into networks of paralogs, and the list of singletons, as well as the gene classification introduced here may represent an univocally defined, reproducible and reliable collection in plant genomics. Indeed, the proposed methodology, which includes several steps of BLAST-based approaches on both protein, gene and intergenic regions, is described with all the details, thus representing a valid support for similar efforts in different contexts. Since the classification and the results we provided can be a reference, as well as the starting point for further analyses in structural and evolutionary genomics, they were made freely accessible through a web resource [80].

Our results highlight the strong need for further investigations on the *A. thaliana* gene annotation and pave the way for novel analyses relevant for plant genomics.
